# Two new abyssal Harpiniinae Barnard & Drummond, 1978 (Amphipoda, Phoxocephalidae) from the Clarion-Clipperton Zone

**DOI:** 10.3897/zookeys.1274.140727

**Published:** 2026-03-24

**Authors:** Luiz F. Andrade, Anna M. Jażdżewska

**Affiliations:** 1 Department of Invertebrate Zoology and Hydrobiology, University of Lodz, Banacha 12/16, 90-237, Lodz, Poland University of Lodz Lodz Poland

**Keywords:** Amphipods, barcoding, COI, deep sea, *
Harpinia
*, *
Harpiniopsis
*, new record, new species, Pacific Ocean, systematics

## Abstract

Two new species of the phoxocephalid subfamily Harpiniinae are described from the easternmost sector of the Clarion-Clipperton Zone. The material examined was collected with epibenthic sledges during the projects ABYSSLINE-2, MANGAN 2016 and MANGAN 2018 from depths ranging from 4133 to 4359 metres. *Harpinia lobata***sp. nov**. is mainly characterized by: head anteroventral corner with a small projection; gnathopod 1 coxa anteroventrally produced; pereopod 6 basis with posterior proximal expanded lobe; and epimeron 3 posteroventral corner rounded. *Harpiniopsis pedro***sp. nov**. can be diagnosed by: head anteroventral corner with a large projection; mandible palp article 2 shorter than article 3; gnathopods 1–2 palm defined by a subacute hump; pereopod 6 basis with a posterior distal lobe reaching the apex of ischium; and epimeron 3 posteroventral corner produced as a slightly curved spine. Here, we provide the deepest record of the known *Harpinia* species and the first *Harpiniopsis* record from the Pacific abyssal plain.

## Introduction

The family Phoxocephalidae G.O. Sars, 1891 comprises primarily benthic species that constitute abundant and diverse components of both shallow-water and deep-sea marine assemblages (Barnard and Drummond 1978; Knox et al. 2012; Jażdżewska 2015; Brix et al. 2018). Study of the benthic fauna of the Clarion-Clipperton Zone (CCZ), a region in the central east Pacific characterized by the presence of manganese nodules, revealed Phoxocephalidae to be highly abundant and consisting of species new to science (Jażdżewska et al. 2025).

Phoxocephalids are divided into two subfamilies, Phoxocephalinae G.O. Sars, 1891 and Harpiniinae Barnard & Drummond, 1978, mainly differentiated by the pereopod 5 basis which is broad in the former and slender in the latter (De Broyer et al. 2007; Alonso de Pina et al. 2008; Andrade and Senna 2019). Harpiniinae comprises 11 genera and is widely distributed geographically and bathymetrically (Barnard and Drummond 1978). Among the harpiniins, *Harpinia* Boeck, 1876 and *Harpiniopsis* Stephensen, 1925 are the most speciose, consisting of 26 and 30 species, respectively (Horton et al. 2024).

Historically, *Harpinia* species have primarily been documented predominantly in the Northern Hemisphere. However, recent studies have extended the range of this genus to the Southern Hemisphere (Andrade et al. 2023). In contrast, *Harpiniopsis* has a more cosmopolitan distribution, with species reported from both hemispheres. Both genera are very similar morphologically, leading to a complicated taxonomic distinction. This has resulted in many taxonomic revisions in the past since male specimens, which are rarely sampled, are crucial for taxonomic differentiation between these genera (Karaman 1993; King et al. 2004; Andrade et al. 2023).

During the examination of phoxocephalid specimens collected in the Clarion-Clipperton Zone, in the North Pacific during the ABYSSLINE-2, MANGAN 2016, and MANGAN 2018 expeditions, two new species of Harpiniinae were identified. One of them is assigned to the genus *Harpinia* and the other to *Harpiniopsis*. The classification of the *Harpinia* species was more precise due to the presence of a male individual showing a short flagellum of antenna 2 and well-developed callynophores forming dense tufts on the peduncle articles of antenna 1. Although males were not available for *Harpiniopsis*, based on Barnard and Karaman (1991), subtle morphological characters of antenna 2 and uropods 1–3 combined make the species pertinent to this genus.

## Material and methods

The material for the present study was sampled in the central east Pacific, specifically in the easternmost sector of the Clarion-Clipperton Zone. The material was collected with an epibenthic sledge (EBS) during three scientific deep-sea cruises: the ABYSSLINE-2 (ABYSSal baseLINE project) in 2015, MANGAN 2016, and MANGAN 2018. For details of gear deployment and sample processing see Jażdżewska and Horton (2026) and Jażdżewska et al. (2025).

Individuals were initially examined using a Nikon SMZ800 stereomicroscope. Appendage photographs were taken using a Nikon Eclipse Ci-L equipped with a Delta Optical DLT-Cam PRO 5MP. The habitus of the holotypes are presented as photographs obtained with a confocal laser scanning microscope (CLSM). The holotypes were stained in Congo red and acid fuchsin, temporarily mounted onto slides with glycerin and examined with a Leica TCS SPV equipped with a Leica DM5000 B upright microscope and three visible-light lasers (DPSS 10 mW 561 nm; HeNe 10 mW 633 nm; Ar 100 mW 458, 476, 488 and 514 nm), combined with the software LAS AF v. 2.2.1 (Leica Application Suite, Advanced Fluorescence). A series of photographic stacks were obtained, collecting overlapping optical sections throughout the whole preparation (Michels and Büntzow 2010; Kamanli et al. 2017).

Chosen specimens were dissected and mounted on permanent slides using polyvinyl-lactophenol containing lignin pink. Photographs from the microscope were used as the basis for line drawings, which were inked with CorelDRAW 2018.

In the descriptions and figures the following abbreviations were used: Hb = habitus; A1, 2 = antenna 1, 2; Ep1–3 = epimera 1–3; Hd = head; L/R Md = left/right mandible; Mx1, 2 = maxilla 1, 2; Mxp = maxilliped; c4 = coxa 4; G1, 2 = gnathopod 1, 2; P3–7 = pereopod 3–7; U1–3 = uropod 1–3; T = telson.

The registered type material is deposited in the Senckenberg Museum (**SMF**; Frankfurt, Germany).

All individuals were subjected to cytochrome *c* oxidase subunit I gene (COI) barcoding prior to the identification of the species. The molecular procedures are described in Jażdżewska et al. (2025). All sequences were deposited in GenBank with the accession numbers: PQ734223, PQ734298, PQ734351, PQ734390, PQ734439, PQ734492 and PQ734519. The relevant voucher information, taxonomic classifications and sequences are deposited in the data set “DS-AMPHICCZ” in the Barcode of Life Data System (BOLD) (https://doi.org/10.5883/DS-AMPHICCZ) (www.boldsystems.org) (Ratnasingham and Hebert 2007).

## Results

### Taxonomy

**Family Phoxocephalidae G.O. Sars, 1891**

**Subfamily Harpiniinae Barnard & Drummond, 1978**

**Genus *Harpinia* Boeck, 1876**

#### Harpinia lobata

Taxon classificationAnimaliaAmphipodaPhoxocephalidae

Andrade & Jażdżewska sp. nov.

288E8851-7944-5F47-A8C3-52B3EA5E28B7

https://zoobank.org/3C74FA4D-A905-47F7-8491-C269FD3D6352

Figs 1, 2, 3, 4

##### Material examined.

***Holotype***: Pacific Ocean • 1 ♀, 6.5 mm; Clarion-Clipperton Zone, 12°21.6'N, 116°42'W; depth 4170 m; 10/03/2015; UKSR-1 exploration contract area, R/V *Thompson*, ABYSSLINE-2, Station AB2-EB09, epibenthic sledge; dissected and drawn; SMF 63349, COI (PQ734223). ***Paratype***: Pacific Ocean • 1 ♂; Clarion-Clipperton Zone, 12°21.6'N, 116°42'W; depth 4170 m depth; 10/03/2015; UKSR-1 exploration contract area; R/V *Thompson*, ABYSSLINE-2, Station AB2-EB09, epibenthic sledge; dissected and drawn; SMF 63348, COI (PQ734351).

##### Type locality.

Abyssal Pacific Ocean, Clarion-Clipperton Zone, 12°21.6'N, 116°42'W, depth 4170 m.

##### Diagnosis.

***Head*** anteroventral corner with a small subacute projection. ***Antenna 2*** peduncle article 4 with two distal facial rows of long setae. ***Mandible*** right *lacinia mobilis* with four spines, left lacinia mobilis apically multicuspidate, palp article 2 stout. ***Gnathopod 1*** coxa anteroventrally produced. ***Pereopod 6*** basis posterior margin with a proximal expanded lobe. ***Pereopod 7*** basis posteroventral margin expanded and weakly serrate. ***Epimeron 3*** posteroventral corner rounded, without any spine or projection. ***Telson*** deeply cleft, with one mid-dorsal plumose seta present on each side.

##### Description.

Based on the female holotype SMF 63349.

**Figure 1.**
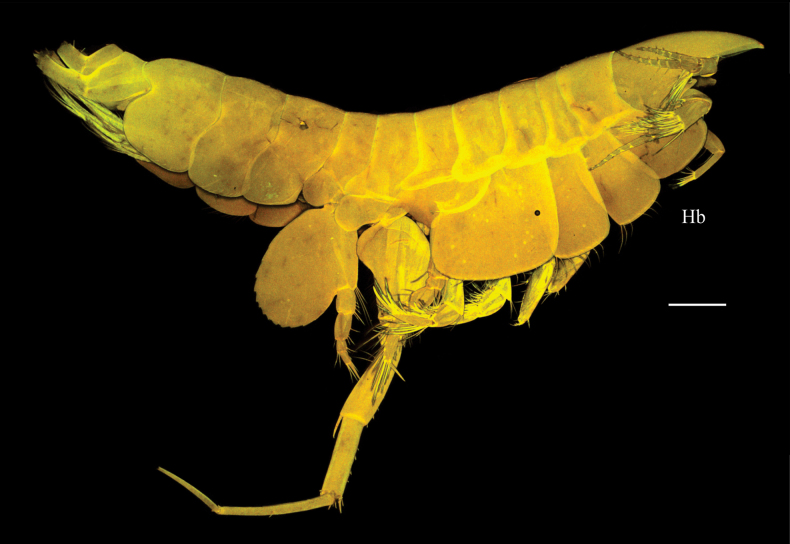
*Harpinia lobata* sp. nov., female holotype SMF 63349. Scale bars: 0.5 mm (Hb).

***Habitus*** as in Fig. 1.

**Figure 2.**
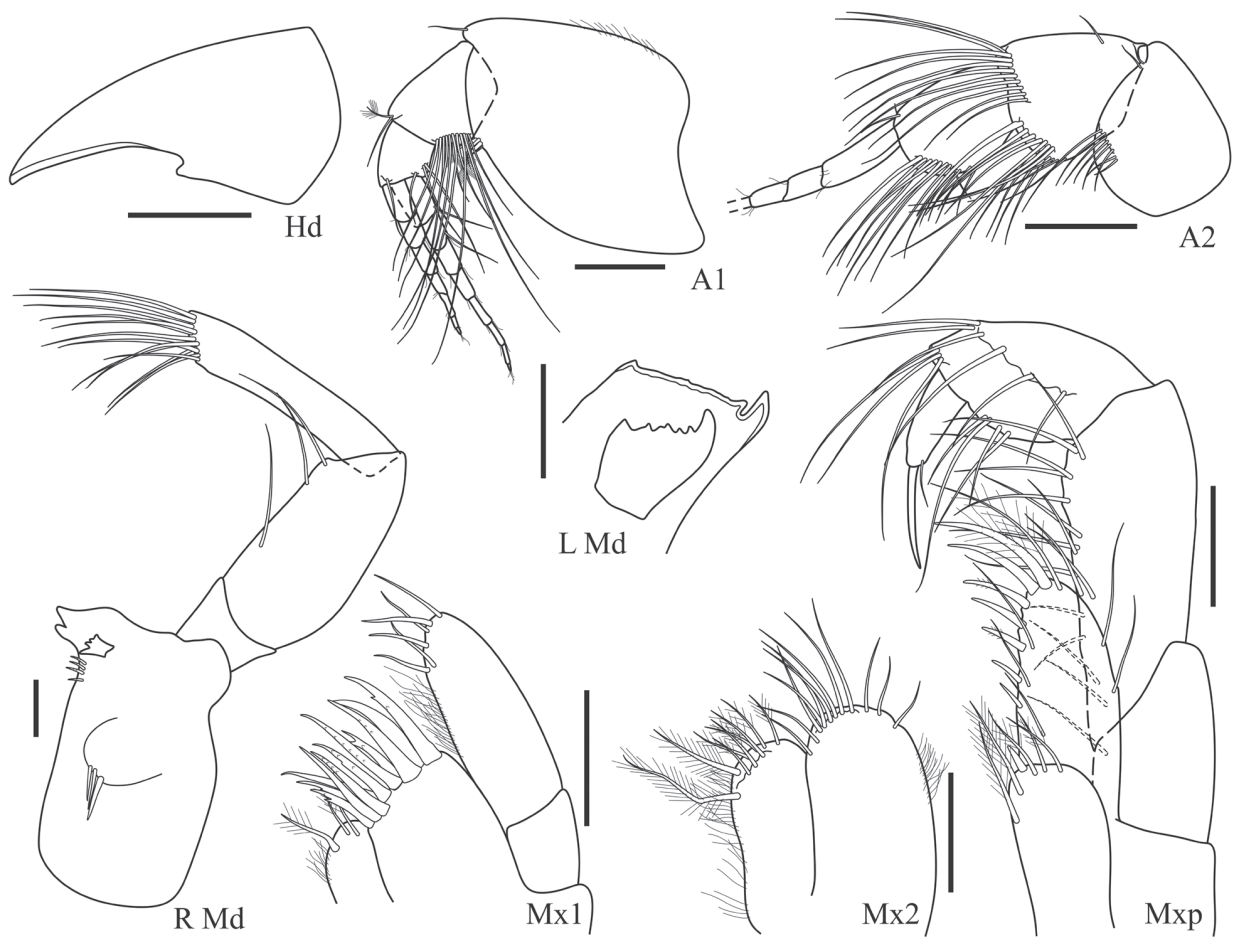
*Harpinia lobata* sp. nov., female holotype SMF 63349. Scale bars: 0.5 mm (Hd); 0.3 mm (A1–2); 0.1 mm (L Md, R Md, Mxp, Mx1–2).

**Head (Fig. 2): *Head*** anteroventral corner with a small subacute projection. ***Antenna 1*** peduncle article 1 slightly longer than wide, ventral margin with few distal setae, article 2 with a facial row of long setae, article 3 short and subquadrate; primary flagellum eight-articulate; accessory flagellum seven-articulate. ***Antenna 2*** peduncle article 4 slightly longer than wide, with two distal facial rows of long setae, being one extending dorsally and the other ventrally, article 5 with a lower facial row of long setae; flagellum damaged, at least three-articulate. ***Mandible*** incisor with a main spine followed by a cutting surface (left) – with two spines (right), lacinia mobilis apically multicuspidate (left) – with four spines (right), molar as a hump with three robust setae, accessory setal row with four robust setae; palp three-articulate, article 2 stout, article 3 apex oblique and setose, as long as article 2. ***Maxilla 1*** inner plate with three apical plumose setae; outer plate with 11 apical robust setae; palp two-articulate, article 2 apex with six setae. ***Maxilla 2*** inner plate subequal to outer, with apical plumose setae; outer plate with apical simple setae. ***Maxilliped*** inner plate with plumose setae and one apical robust seta; outer plate with mesial robust setae increasing in length towards the apex; palp articles 2–3 weakly setose, article 4 about half the length of article 3, bearing a long, embedded nail.

**Figure 3.**
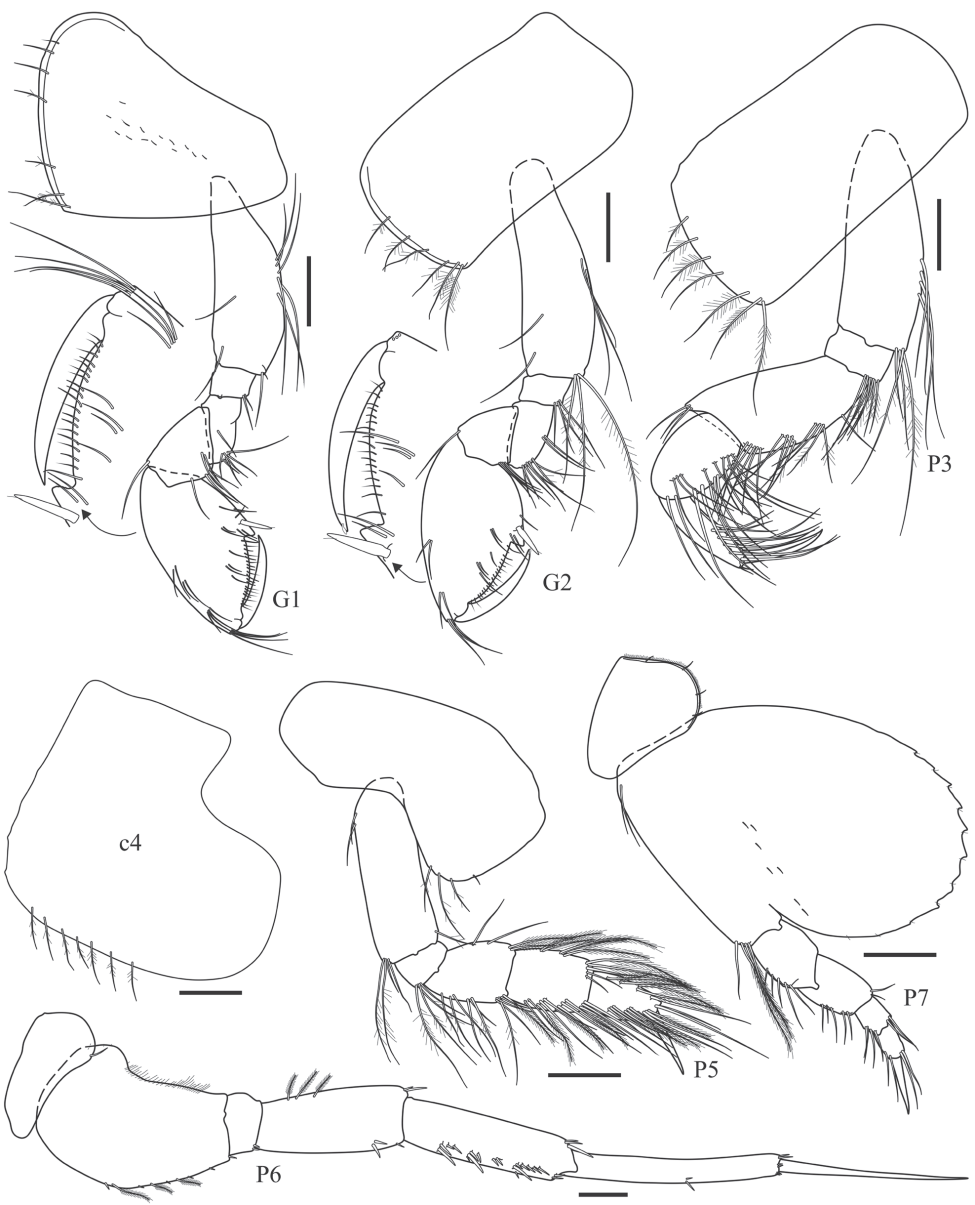
*Harpinia lobata* sp. nov., female holotype SMF 63349. Scale bars: 0.3 mm (G1–2, P3–7).

**Pereon (Fig. 3): *Gnathopod 1*** coxa anteroventrally produced, with sparse ventral pappose/simples setae; basis to carpus ordinary, weakly setose; propodus 1.9× longer than wide, posterior margin with a robust seta near palmar corner, palm acute, defined by an excavation forming a medium palmar hump; dactylus as long as palm. ***Gnathopod 2*** coxa with ventral pappose setae; carpus shorter than in gnathopod 1; propodus very similar to gnathopod 1 but slightly broader. ***Pereopod 3*** coxa with ventral pappose setae; merus 1.8× longer than wide, ventral margin with three sets of simple/pappose setae; propodus distally setose, 3.8× longer than wide; dactylus about 75% the length of propodus. ***Pereopod 4*** coxa posteroventral lobe strongly expanded, posterodorsal margin exacavate, ventral margin with pappose setae; other articles very similar to pereopod 3. ***Pereopod 5*** coxa bilobate, posterior lobe with pappose setae; basis 3.1× longer than wide; merus weakly setose; carpus and propodus both margins setose with plumose setae; dactylus as long as propodus. ***Pereopod 6*** coxa posterior margin weakly expanded, with one seta; basis posterior margin with a proximal expanded lobe; carpus anterior margin with sets of robust setae; dactylus long, slightly shorter than propodus. ***Pereopod 7*** coxa subtriangular, posterior margin with four setae; basis slightly longer than wide, posteroventral margin expanded and weakly serrate; dactylus as long as carpus and propodus combined.

**Figure 4.**
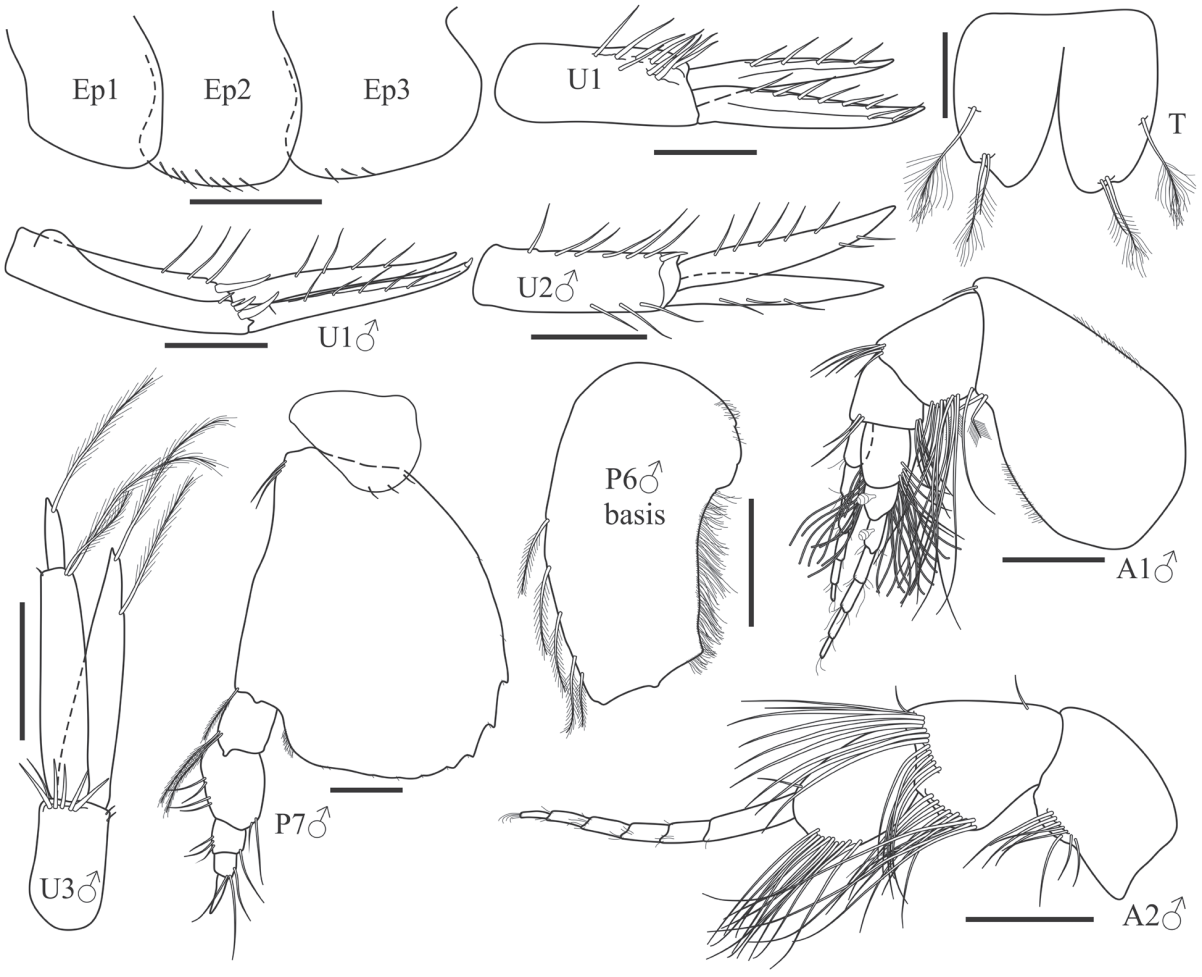
*Harpinia lobata* sp. nov., female holotype SMF 63349, male paratype SMF 63348. Scale bars: 0.5 mm (Ep1–3); 0.3 mm (A1–2, U1–3, P6–7); 0.1 mm (T).

**Pleon (Fig. 4): *Epimeron 1*** without setae. ***Epimera 2–3*** ventral margin with simple setae, posteroventral corner rounded, without any spine or projection. ***Uropod 1*** peduncle 2.6× longer than wide, with dorsal setae; rami subequal in length, without apical nail, bearing dorsal robust setae. ***Telson*** deeply cleft, as long as wide, with one mid-dorsal and two apical plumose setae.

Sexually dimorphic characters (Fig. 4). Based on the male paratype SMF 63348.

***Antenna 1*** with well-developed callynophores forming dense tufts on peduncle articles 2–3.

***Pereopod 6*** basis posterior margin with a proximal expanded lobe, posterior margin with a dense row of setules. ***Pereopod 7*** merus broad.

***Uropod 1*** peduncle 4.4× longer than wide, bearing simple dorsal setae and robust setae distally; rami subequal in length, with dorsal setae; outer ramus with an apical embedded nail. ***Uropod 2*** peduncle 3.3× longer than wide, bearing dorsal setae; rami subequal in length, with dorsal setae. ***Uropod 3*** peduncle with five distal robust setae; rami subequal in length; outer ramus article 1 with two distal plumose setae, article 2 with one apical plumose seta; inner ramus slightly longer than article 1 of outer ramus, with two distal plumose setae.

##### Molecular identification.

Following the definition given by Pleijel et al. (2008), the sequence of the female holotype of *Harpinia lobata* sp. nov. (SMF 63349, GenBank accession number PQ734223) is designated as a hologenophore of all obtained sequences. The sequence of the paratype of the species is deposited in GenBank with the following accession number: PQ734351. The species has also received a Barcode Index Number from Barcode of Life Data Systems: BOLD:AEA9473 (https://doi.org/10.5883/BOLD:AEA9473).

##### Distribution.

Only known from the type locality.

##### Etymology.

The specific epithet *lobata*, from the Latin “lobatus” (lobed), is given due to the prominent posterior lobe on the pereopod 6 basis.

##### Remarks.

*Harpinia lobata* sp. nov. is morphologically similar to *Harpinia curtipes* Stephensen, 1925 and *Harpinia truncata* G.O. Sars, 1891 as they all present the following: epimeron 3 posteriorly rounded, without any projection or spine; pereopod 6 dactylus shorter than propodus; and pereopod 7 basis anteroventral corner not produced, posteroventral margin crenulate/serrate, without large spines. However, the new species can be mainly distinguished from them due to the following: gnathopod 1 coxa anteroventrally produced (vs. not produced in both species); and pereopod 6 basis posterior margin with a proximal expanded lobe (vs. without proximal lobe in both species).

#### Harpiniopsis pedro

Taxon classificationAnimaliaAmphipodaPhoxocephalidae

Andrade & Jażdżewska sp. nov.

7365BFD0-D1E1-5813-ACA9-2D67AAD8E05C

https://zoobank.org/F117A725-7D8F-4510-B955-E2E0B272F269

Figs 5, 6, 7, 8

##### Material examined.

***Holotype***: Pacific Ocean • 1 ♀, 5.7 mm; Clarion-Clipperton Zone; 11°47.862'N, 117°30.639'W – 11°47.152'N, 117°29.490'W; depth 4359–4356 m; 9/05/2016; BGR exploration contract area, R/V *Kilo Moana*, MANGAN 2016, Station Ma 16-95, epibenthic sledge; dissected and drawn; SMF 63354, COI (PQ734298). ***Paratype***: Pacific Ocean • 2 ♀; Clarion-Clipperton Zone, BGR exploration contract area; 11°47.862'N, 117°30.639'W – 11°47.152'N, 117°29.490'W; depth 4359–4356 m; 9/05/2016; BGR exploration contract area, R/V *Kilo Moana*, MANGAN 2016; Station Ma 16-95, epibenthic sledge; SMF 63352, COI (PQ734439), SMF 63353, COI (PQ734519). • 1 ♀; Clarion-Clipperton Zone; 11°47.436'N, 117°32.213'W – 11°47.677'N, 117°30.910'W; depth 4351–4352 m; 9/05/2018; BGR exploration contract area, R/V *Sonne* ; Station SO 262-155, epibenthic sledge; SMF 63350, COI (PQ734390) • 1 ♀; Clarion-Clipperton Zone; 11°49.654'N, 117°00.299'W – 11°49.902'N, 116°59.174'W; depth 4133–4143 m; 1/05/2016; BGR exploration contract area, R/V *Kilo Moana*, MANGAN 2016; Station Ma 16-28, epibenthic sledge; SMF 63351, COI (PQ734492).

##### Type locality.

Abyssal Pacific Ocean, Clarion-Clipperton Zone, 11°47.862'N, 117°30.639'W – 11°47.152'N, 117°29.490'W, depth 4359–4356 m.

##### Diagnosis.

***Head*** anteroventral corner with a large acute projection. ***Mandible*** right lacinia mobilis bifid, left lacinia mobilis with four teeth, palp article 2 longer than article 3, with an inner medial long pappose setae. ***Gnathopods 1–2*** palm defined by a subacute hump. ***Pereopod 6*** basis posterior margin with a distal lobe reaching the distal apex of ischium. ***Pereopod 7*** posteroventral margin expanded and serrate. ***Epimeron 3*** posteroventral corner produced as a slightly curved spine. ***Uropod 2*** rami naked. ***Telson*** deeply cleft, with two mid-dorsal plumose setae present on each side.

##### Description.

Based on the female holotype (SMF 63354).

**Figure 5.**
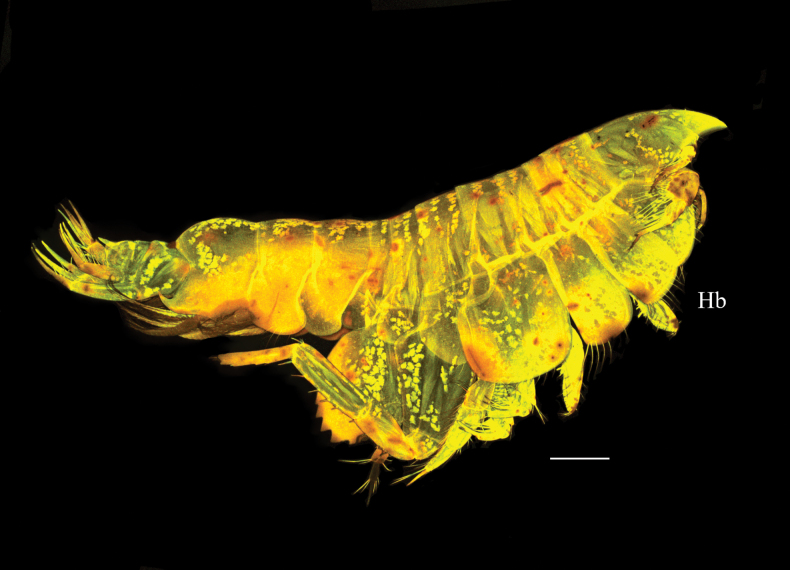
*Harpiniopsis pedro* sp. nov., female holotype SMF 63354. Scale bars: 0.5 mm (Hb).

***Habitus*** as in Fig. 5.

**Figure 6.**
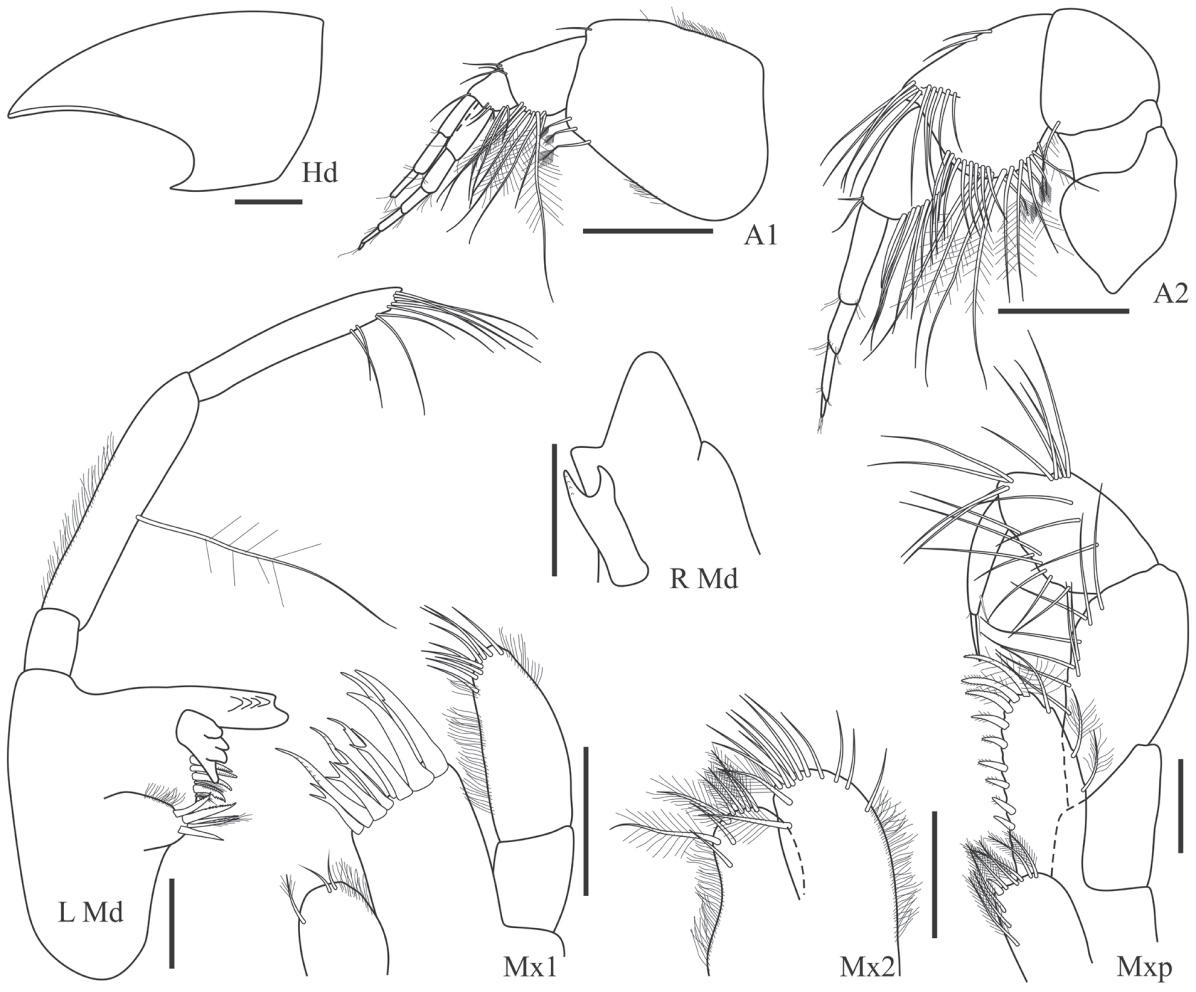
*Harpiniopsis pedro* sp. nov., female holotype SMF 63354. Scale bars: 0.1 mm (L Md, Mxp, Mx1–2); 0.3 mm (Hd, A1–2); 0.05 mm (R Md).

**Head (Fig. 6): *Head anteroventral corner*** with a large acute projection. ***Antenna 1*** peduncle article 1 about 1.3× longer than wide, ventral margin with three distal brush setae, article 2 ventral margin with row of long pappose setae; article 3 short and subquadrate; primary flagellum 6-articulate; accessory flagellum 3-articulate. ***Antenna 2*** peduncle article 1 not ensiform, article 4 setose, with two facial rows of setae dorso-distally, article 5 ventral margin with a row of long setae; flagellum 4-articulate. ***Mandible*** incisor with six spines (left) – with three spines (right), lacinia mobilis with four teeth (left) – bifid (right), molar as a hump with one plumose and four robust setae; palp 3-articulate, article 2 with an inner medial long pappose setae, article 3 apex oblique and setose, 0.8× the length of article 2. ***Maxilla 1*** inner plate with three apical setae; outer plate with 10 apical robust setae; palp 2-articulate, article 2 apex setose. ***Maxilla 2*** inner plate subequal to outer, with apical plumose setae; outer plate with lateral and apical simple setae. ***Maxilliped*** inner plate with plumose setae and one apical robust seta; outer plate with mesial robust setae increasing in length towards the apex; palp articles 2–3 weakly setose, article 4 about 0.7× the length of article 3, bearing a medium embedded nail.

**Figure 7.**
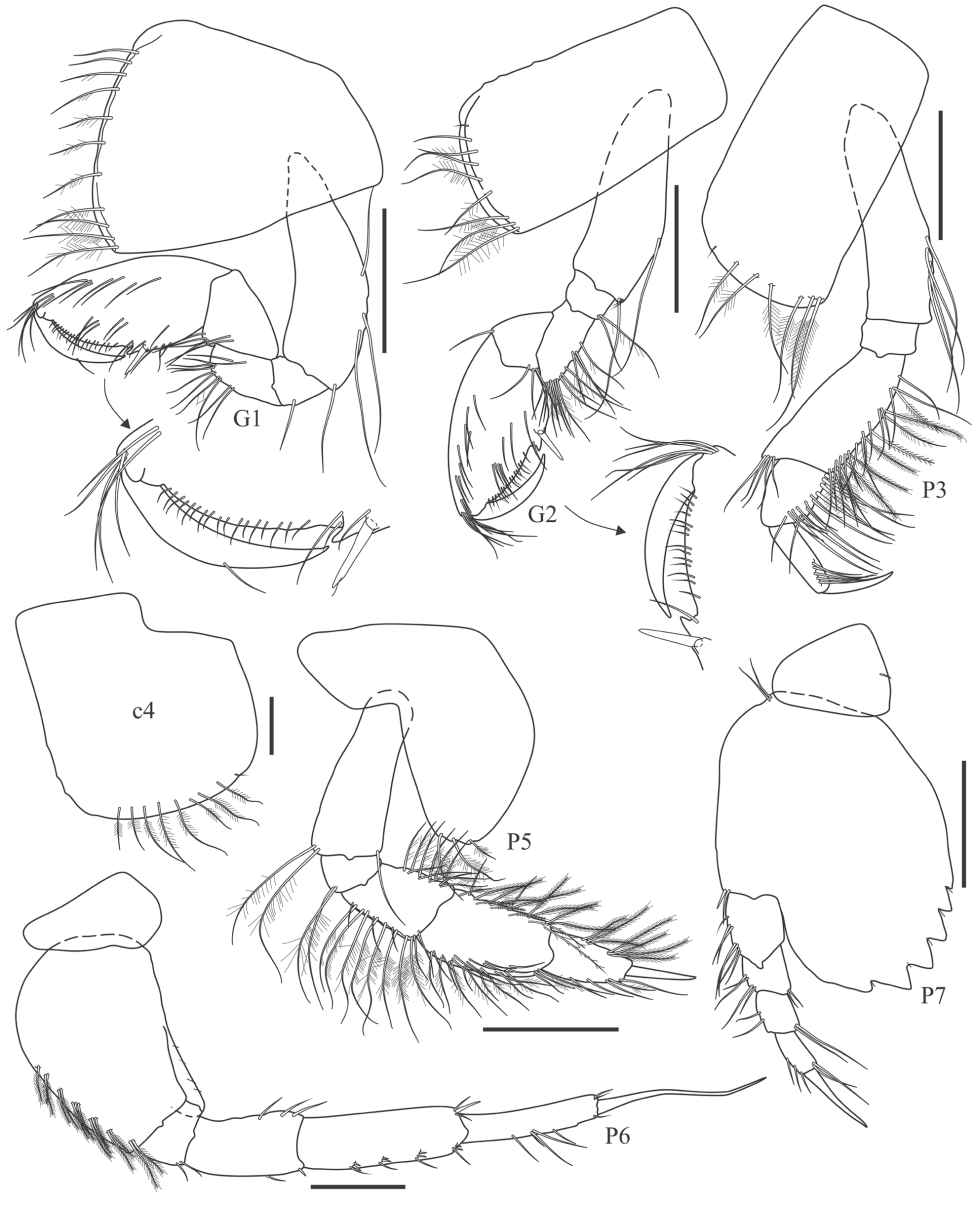
*Harpiniopsis pedro* sp. nov., female holotype SMF 63354. Scale bars: 0.5 mm (G1–2, P3, P5–7); 0.3 mm (c4).

**Pereon (Fig. 7): *Gnathopod 1*** coxa with ventral pappose/simple setae; basis to carpus ordinary, weakly setose; propodus 2× longer than wide, posterior margin with a robust seta near palmar corner, palm acute, defined by a subacute hump; dactylus as long as palm. ***Gnathopod 2*** coxa with ventral pappose/simple setae; carpus shorter than in gnathopod 1; propodus very similar to gnathopod 1 but with palm defined by an acute hump. ***Pereopod 3*** coxa with ventral pappose setae; merus 2.2× longer than wide, ventral margin moderately setose with simple and plumose setae; propodus with setae, 3.3× longer than wide; dactylus about 85% the length of propodus. ***Pereopod 4*** coxa posteroventral lobe strongly expanded, posterodorsal margin exacavate, ventral margin with pappose setae; other articles very similar to pereopod 3. ***Pereopod 5*** coxa bilobate, posterior lobe with pappose setae; merus to propodus anterior margin with long pappose setae, posterior margin with plumose setae; dactylus 80% the length of propodus. ***Pereopod 6*** coxa subtriangular, without setae; basis posterior margin with a distal lobe reaching the distal apex of ischium, anterior margin with sets of plumose setae; carpus anterior margin with sets of robust setae; dactylus long, 1.2× longer than propodus. ***Pereopod 7*** coxa subtriangular; basis 1.2× longer than wide, posteroventral margin expanded and serrate; dactylus as long as carpus and propodus combined.

**Figure 8.**
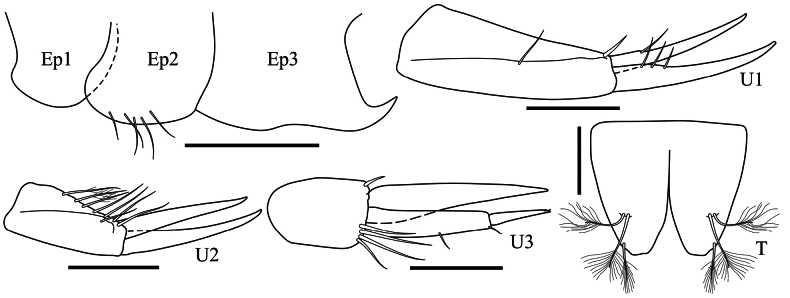
*Harpiniopsis pedro* sp. nov., female holotype SMF 63354. Scale bars: 0.5 mm (Ep1–3); 0.3 mm (U1–3); 0.1 mm (T).

**Pleon (Fig. 8): *Epimeron 1*** without setae. ***Epimeron 2*** ventral margin with simple setae. ***Epimeron 3*** posteroventral corner produced as a slightly curved spine. ***Uropod 1*** peduncle 2.8× longer than wide; rami subequal in length, without apical nail, with spart dorsal setae. ***Uropod 2*** peduncle 2.3× longer than wide; rami subequal in length, naked, without apical nail. ***Uropod 3*** peduncle with five distal robust setae; rami equal in length, almost naked. ***Telson*** slightly wider than long, with two mid-dorsal and one apical plumose seta.

##### Molecular identification.

Following the definition given by Pleijel et al. (2008), the sequence of the female holotype of *Harpiniopsis pedro* sp. nov. (SMF 63354, GenBank accession number PQ734298) is designed as a hologenophore of all obtained sequences. The sequences of the paratypes of the species are deposited in GenBank with the following accession numbers: PQ734390, PQ734439, PQ734492 and PQ734519. The species has also received a Barcode Index Number from Barcode of Life Data Systems: BOLD:AEB4266 (https://doi.org/10.5883/BOLD:AEB4266).

##### Distribution.

Abyssal Pacific Ocean, Clarion-Clipperton Zone, 4133–4359 m.

##### Etymology.

The specific epithet *pedro*, used as a noun in apposition, is given in honor of Prof. Dr Pedro Mart**í**nez Arbizu from the German Center for Marine Biodiversity Research (DZMB), Senckenberg am Meer, renowned marine biologist and a good friend of the junior author who kindly offered the CCZ amphipod collection to study and was first who suggested organizing taxonomic description workshop that resulted in multiple amphipod species descriptions.

##### Remarks.

*Harpiniopsis pedro* sp. nov. is morphologically similar to *Harpiniopsis australis* (J.L. Barnard, 1961) and *Harpiniopsis wandichia* (J.L. Barnard, 1962) as they present the following: head anteroventral corner with an acute projection; pereopod 7 basis with a similar posteroventral serrate arrangement, reaching the distal apex of merus; epimeron 3 posteroventral corner produced into a spine.

However, the new species can be mainly distinguished from *Harpiniopsis australis* by the following (characters of *Harpiniopsis australis* between parentheses): pereopod 6 basis distal lobe reaching the distal apex of ischium (vs. reaching half of ischium), dactylus longer than propodus (vs. as long as propodus); pereopod 7 basis posteroventral projections broader about 1.2× as broad as long and bearing both subacute and blunt projections (vs. narrower about 0.8× as broad as long and bearing acute projections); epimeron 3 posteroventral spine slightly curved and of moderate size (vs. strongly curved and large); and telson with mid-dorsal setae (vs. without), apex truncate (vs. acute).

Also, *Harpiniopsis pedro* sp. nov. differs from *Harpiniopsis wandichia* by the following (characters of *Harpiniopsis wandichia* in parentheses): head anteroventral corner with large acute projection (vs. small); pereopod 5 merus to propodus anterior margin setose (vs. weakly setose); and telson with mid-dorsal setae (vs. without), apex truncate (vs. subacute).

## Discussion

Until present, eight of the 26 known *Harpinia* species were reported from depths below 2000 m and they are: *Harpinia curtipes* Stephensen, 1925, *Harpinia dellavallei* Chevreux, 1910, *Harpinia mucronata* G.O. Sars, 1879, *Harpinia pico* Bellan-Santini, 2007, *Harpinia plumosa* (Krøyer, 1842), *Harpinia propinqva* G.O. Sars 1891, *Harpinia serrata* G.O. Sars 1879 and *Harpinia truncata*. All these species, excluding *Harpinia mucronata*, were recorded from the Atlantic Ocean or the Mediterranean Sea (Barnard 1962; Bellan-Santini 1985; Brandt 1997; Bellan-Santini 2007; Budaeva et al. 2008; Bergmann et al. 2011). *Harpinia mucronata* was reported by Blake et al. (2009) from the San Francisco Deep Ocean Disposal Site, California from a depth range of 2160–3142 m. However, considering this species being described and commonly recorded from the North Atlantic the identification shall be treated with caution. Moreover, none of the above-cited species was found at abyssal (below 4000 m) depths, so the present finding of *Harpinia lobata* sp. nov. at the depth of 4170 m provides the deepest record of the known *Harpinia* species.

In the case of *Harpiniopsis* as many as 12 species were reported from depths below 2000 m. Of them, *Harpiniopsis capensis* (J.L. Barnard, 1962) and *Harpiniopsis wandichia* were found in the Atlantic Ocean, with the first collected at abyssal depths (4961–4983 m) (Barnard 1962). The group of the Pacific deep-sea *Harpiniopsis* species consists of: *Harpiniopsis emeryi* J.L. Barnard, 1960, *Harpiniopsis fulgens* J.L. Barnard, 1960, *Harpiniopsis galera* J.L. Barnard, 1960, *Harpiniopsis naiadis* J.L. Barnard, 1960, *Harpiniopsis orientalis* (Bulycheva, 1936), *Harpiniopsis pacifica* (Bulycheva, 1936), *Harpiniopsis percellaris* J.L. Barnard, 1971, *Harpiniopsis profundis* J.L. Barnard, 1960, *Harpiniopsis spaercki* (Dahl, 1959), and *Harpiniopsis triplex* J.L. Barnard, 1971. Among these taxa, *Harpiniopsis spaercki* was recorded from the hadal zone (7335–7340 m in the Banda Sea Trench) (Kamenskaya 1981) whereas all remaining were not recorded below 3000 m (Barnard 1967, 1971; Blake et al. 2009; Golovan et al. 2013). The description of *Harpiniopsis pedro* sp. nov. acts as the first record of the genus from the Pacific abyssal plain.

## Supplementary Material

XML Treatment for Harpinia lobata

XML Treatment for Harpiniopsis pedro

## Additional information

### Author ORCIDs

Luiz F. Andrade https://orcid.org/0000-0002-4868-737X

Anna M. Jażdżewska https://orcid.org/0000-0003-2529-0641

### Data availability

All of the data that support the findings of this study are available in the main text.
